# Role of PARP inhibitors beyond BRCA mutation and platinum sensitivity in epithelial ovarian cancer: a meta-analysis of hazard ratios from randomized clinical trials

**DOI:** 10.1186/s12957-023-03027-4

**Published:** 2023-05-23

**Authors:** Roli Purwar, Rakesh Ranjan, Manjusha Pal, Satyanshu K. Upadhyay, Tarun Kumar, Manoj Pandey

**Affiliations:** 1 Department of Surgical Oncology, Institute of Medical Sciences, Banaras Hindu University, Varanasi, 221005 India; 2 Department of Science and Technology, Centre for Interdisciplinary Mathematical Sciences, Banaras Hindu University, Varanasi, 221005 India; 3 Department of Statistics, Banaras Hindu University, Varanasi, 221005 India

## Abstract

**Background:**

PARP inhibitors (PARPi) have a well-established role in platinum-sensitive ovarian cancer (PSOC), in BRCA mutant (BRCAm), and homologous recombination deficiency (HRD) population. However, their role in wild type and homologous recombination proficient population is still not clear.

**Methods:**

A meta-analysis of hazard ratios (HR) of randomized control trials (RCTs) was conducted to study the role of PARPi. The published RCTs comparing the efficacy of PARP inhibitors alone or in combination with chemotherapy and/or target therapies versus placebo/chemotherapy alone/target therapy alone in primary or recurrent ovarian cancer settings were selected. Progression-free survival (PFS) and overall survival (OS) were the primary endpoints.

**Results:**

A total of 14 primary studies and 5 updated studies are considered, consisting of 5363 patients. Overall, HR for PFS was 0.50 [95% CI 0.40–0.62]. HR of PFS was 0.94 [95% CI 0.76–1.15] in the PROC group, 0.41 [95% CI 0.29–0.60] was in HRD with BRCA unknown (BRCAuk), 0.38 [95% CI 0.26–0.57] in HRD with BRCAm, and 0.52 [95% CI 0.38–0.71] in HRD with BRCAwt. In the HRP group, overall HR for PFS was 0.67 [95% CI 0.56–0.80], 0.61 [95% CI 0.38–0.99] in HRD unknown with BRCA wt, and 0.40 [95% CI 0.29–0.55] in BRCAm HR for PFS. Overall, HR for OS was 0.86 [95% CI 0.73–1.031].

**Conclusions:**

The results suggest that PARPi have a meaningful clinical benefit in PSOC, HRD, BRACm, and also in HRP and PROC; however, the evidence is not sufficient to recommend their routine use and further studies are needed to expand their role in the HRP and PROC groups.

**Supplementary Information:**

The online version contains supplementary material available at 10.1186/s12957-023-03027-4.

## Introduction

Epithelial cancer of the ovary ranks fifth in cancer death among women and is the seventh most frequent cancer diagnosed worldwide. According to the American Cancer Society, about 19,880 women will receive a new diagnosis of ovarian cancer and about 12,810 women will die from ovarian cancer in the USA in 2022 [1, 2]. The main goal of treatment is to achieve disease control with no residual disease, either by cytoreductive surgery or platinum-based chemotherapy, In case of recurrence, if the disease recurs more than 6 months after giving platinum-based therapy, called as partially platinum-sensitive (between 6 and 12 months), or platinum-sensitive (more than 12 months), it can be rechallenged with the platinum analog [2]. PARP (poly ADP ribose polymerase) protein is a group of intranuclear enzymes that have an integral role in a single-stranded DNA break repair along with many intranuclear activities. If PARP enzymes are blocked by PARP inhibitors, then a single-stranded DNA break is converted into a double-stranded DNA break, which is then repaired by homologous recombination repair (HRR) pathway which is the major pathway of a double-stranded DNA repair [3]. If the patient is HR pathway-deficient (HRD), like having mutation in BRCA1 or BRCA 2 genes, they become more responsive to PARP inhibitor therapy by the process of synthetic lethality. Other HR pathway gene mutations like ATM, CHEK, BRIP2, BALD 1, PALB 2, and RAD 51C in cells also make them sensitive to double-strand break repair drugs, which is the phenomenon being called as BRCAness [4]. Currently, only 3 PARP inhibitors are FDA-approved in ovarian cancer of which Olaparib and Niraparib monotherapy is approved for maintenance in post-primary and recurrent chemotherapy, while Rucaparib for maintenance setting in recurrent ovarian cancer [5].

There are numbers of randomized controlled trial and meta-analysis which had shown the importance of PARP inhibitors in advanced platinum-sensitive ovarian cancer in BRCA-mutant cohort and HRD population in first-line maintenance settings, in recurrent maintenance settings, and in treatment of refractory ovarian cancer after failure of 2nd or 3rd lines of platinum-based chemotherapy [6–8]. The question of using PARP inhibitors in BRCA wild type (BRCAwt) population and homologous recombination-proficient groups (HRP) remains unanswered. PARP inhibitors are more active in platinum-sensitive patients because of similar mechanism of action; however, the question of their role in platinum resistant ovarian cancer needs to be answered.

We conducted this meta-analysis of hazard ratios of randomized control trials to study the role of PARP inhibitors in epithelial cancer of the ovary in platinum-resistant and HRP patients with ovarian cancer.

## Material and methods

### Search strategy, selection, and inclusion criteria

Meta-analysis of phase 2 or 3 randomized controlled trials was performed, and the results are presented according to the Preferred Reporting Items for Systematic Reviews and Meta-Analysis (PRISMA). Articles published between 1987 and June 2022 were considered.

A systematic literature search of PubMed, Embase, and Cochrane library was carried out to identify all published phase 2/3, RCT using the following search strings: (("poly adp ribose polymerase inhibitors"[Pharmacological Action] or "poly adp ribose polymerase inhibitors"[MeSH Terms] or ("poly adp ribose"[All Fields] and "polymerase"[All Fields] and "inhibitors"[All Fields]) or "poly adp ribose polymerase inhibitors"[All Fields] or ("parp"[All Fields] and "inhibitors"[All Fields]) or "parp inhibitors"[All Fields]) and ("ovarian neoplasms"[MeSH Terms] or ("ovarian"[All Fields] and "neoplasms"[All Fields]) or "ovarian neoplasms"[All Fields] or ("ovarian"[All Fields] and "cancer"[All Fields]) OR "ovarian cancer"[All Fields])), and (randomizedcontrolledtrial[Filter]). The last search was performed in June 2022.

Out of 66 studies retrieved, 14 studies were considered for quantitative analysis after the elimination of duplicates and exclusion, and 5 additional studies obtained through hand search were also considered which were updated analysis from the previous studies [6, 7, 9–25]. Manual back reference checks were done and resulted in addition of no further articles.

### Inclusion criteria

All randomized trials that compared the efficacy of PARP inhibitors alone or in combination with chemotherapy and/or target therapies versus placebo/chemotherapy alone/target therapy alone in primary or recurrent ovarian cancer setting are the inclusion criteria.

### Exclusion criteria

All non-randomized studies, retrospective studies, review articles, cohort, observational studies, non-published literature, and abstract presented as part of meetings are the xclusion criteria.

### Data extraction

Two authors (RP and TK) independently scanned all the abstracts and shortlisted the studies meeting the above inclusion criteria, and the data was entered in predefined proforma on an Excel sheet, the following values were recorded: first author information, publication year, clinical trial acronym, country, base line characteristics, study design, inclusion and exclusion criteria, sample size, HRD and BRCA-mutated status, progression-free survival, overall survival, time to first subsequent therapy or death, time to second subsequent therapy, and adverse event.

Any discrepancies were settled after discussion with the third author (MP). Quality assessment was performed using Jadad’s score (Table 1) [26], and the risk of bias was assessed using revised Cochrane risk of bias tool (ROB 2.0 _IRPG_ beta v8) [27] (supplementary file 1). Heterogeneity was assessed using *I* ^2^. Random effect models were used when heterogeneity was high. Forest and funnel plot were prepared. Publication bias was assessed.

**Table 1:** Baseline characteristics of the included studies and Jadad score

Author name and year	Trial name	Design	Settings	Phase	Total number of patients	Case	Control	BRCAm		HRD, BRCAm		HRD, BRCA uk		HRD, BRCAwt		HRP		HRDuk, BRCAwt		PROC		JADADE SCORE R + B + D
								Case	Control	Case	Control	Case	Control	Case	Control	Case	Control	Case	Control	Case	Control	
Penson et al. 2020 [19]	SOLO 3	Olaparib 600 mg vs single-agent chemotherapy	Treatment in recurrent PSOC	3	266	178	88	178	88													2 + 1 + 1
Pujade et al. 2017 [21]	SOLO 2	Olaparib 300 mg vs Placebo	Maintenance in recurrent PSOC	3	295	196	99	196	99													2 + 2 + 1
Moore et al. 2018 [7]	SOLO 1	Olaparib 600 mg vs placebo	Maintenance in primary advanced PSOC	3	391	260	131	260	131													2 + 2 + 1
Coleman et al. 2019 [6]	VELIA	PC + veliparib 300 mg or PC + veliparib 300 mg + veliparib maintainace vs PC + placebo + placebo maintainence	Treatment followed by maintenance in primary advanced PSOC	3	1140	383/382	375	108	92			214	207	106	115	125	124	245	254			1 + 2 + 1
Wu et al. 2021 [25]	NORA	Niraparib 300 mg vs placebo	Maintenance in recurrent PSOC		265	177	88	177	88													2 + 2 + 1
González-Martín et al. 2019 [11]	PRIMA	Niraparib 300 mg or 200 mg vs placebo	Maintenance in primary advanced PSOC	3	733	487	246			152	71			95	55	169	80					1 + 2 + 1
Coleman et al. 2017 [10]	ARIEL 3	Rucaparib 1200 mg vs placebo	Maintenance in recurrent PSOC	3	564	375	189	130	66			236	118	106	52	107	54	32	17			2 + 2 + 1
Vanderstichele et al. 2022 [23]	CLIO	Olaparib 600 mg vs physicians choice CT	Treatment in recurrent PSOC and PROC	3	160	107	53	18	4									89	49	67	33	1 + 0 + 1
Kristeleit et al. 2022 [13]	ARIEL 4	Rucaparib 1200 mg vs CT	Treatment in recurrent PSOC and PROC	3	349	233	116	233	116											110	51	2 + 0 + 1
Colombo et al. 2022 [24]	BAROCCO	Olaparib + cediranib(inttermittent/continous) vs paclitaxel in PROC	Treatment in recurrent PROC	2	123	82	41															2 + 0 + 1
Oza et al. 2015 [18]		Olaparib + pc f/b olaparib vs pc f/b placebo	Treatment f/b Maintenance in recurrent PSOC	2	162	81	81	20	21													2 + 0 + 1
Lederman et al. 2012 [14]		Olaparib 800 mg vs placebo	Maintenance in recurrent PSOC	2	265	136	129	74	62									57	61			2 + 2 + 1
Kaye et al. 2012 [12]		Olaparib 400 mg or 800 mg vs pegylated liposomal doxorubicin	Maintenance in recurrent PSOC	2	97	64	33															2 + 0 + 1

*PC *Paclitaxel + carboplatin, *PROC *platinum-resistant ovarian cancer, *PSOC *platinum-sensitive ovarian cancer, *BRCAm *BRCA mutated, *BRCA wt *BRCA wild type, *HRD* homologous recombination-deficient, *HRP *homologous recombination proficient, *BRCAuk *BRCA status unknown, *HRDuk* HRD status unknown, *R* + *B* + *D *randomization + blinding + details of drop outs

As variables evaluated in different studies were different, only the variables common between various studies were considered for the final analysis.

### Outcomes of interest and definitions

For the final analysis, two primary endpoints were considered, progression-free survival (PFS) and overall survival (OS). The secondary endpoints were PFS 2, TFST, and TSST. The OS was defined as the time from the date of recruitment to death, and PFS was defined as the time from recruitment to the progression of the disease as described in the studies. PFS 2 was the time from randomization to the second progression of the disease, TFST from randomization to the first subsequent therapy or death, and TSST the time to the second subsequent therapy or death.

### Statistical analysis

As the data for individual patients were not available, meta-analysis of hazard ratio was carried out as described by Tierney et al. [28]. The package “meta,” of statistical software R, was used to perform the meta-analysis [29]. The program is enclosed as Additional file 2. The method described by Purwar et al., was used [30].

The manuscript is presented following the Preferred Reporting Items for Systematic Reviews and Meta-Analyses (PRISMA) guidelines [31], and the checklist is submitted (Additional file 3). The study was registered in PROSPERO with registration number CRD42022310206 [32].

## Results

**Fig. 1:**
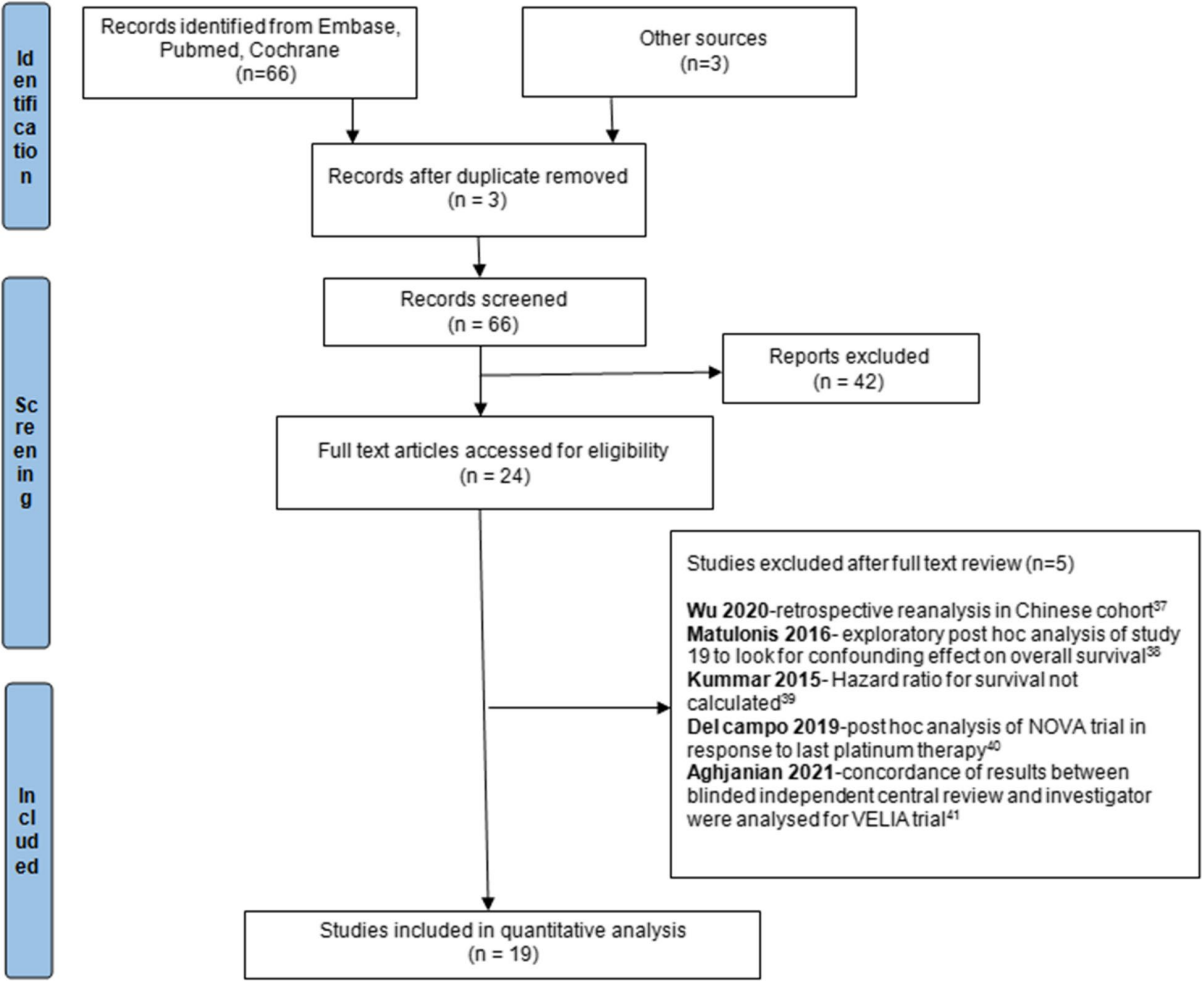
Flow chart showing the study selection and reasons for the excluded studies [33–37]

**Fig. 2:**
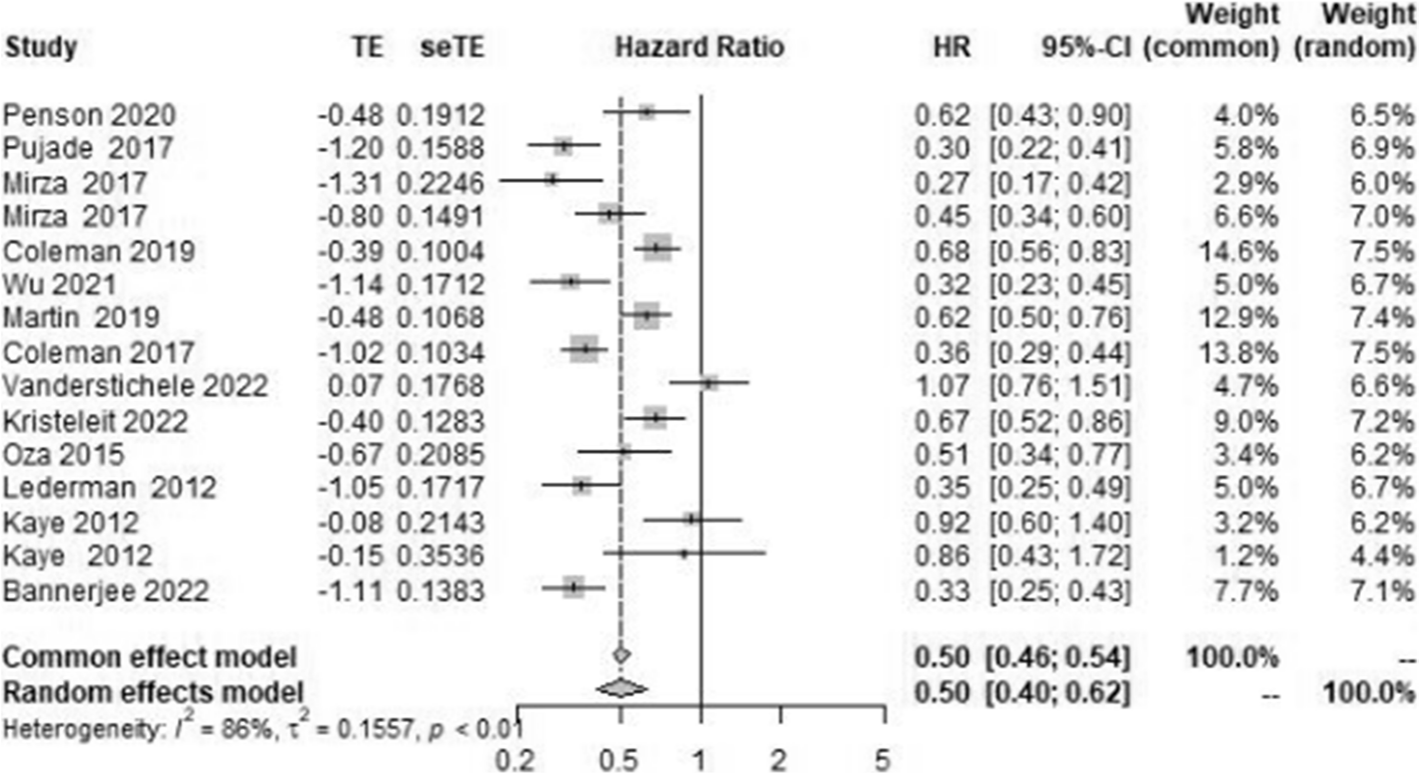
Forest plot showing progression-free survival in PARP inhibitor arm (case) and chemotherapy/placebo arm in platinum-sensitive ovarian cancer (control)

**Fig. 3:**
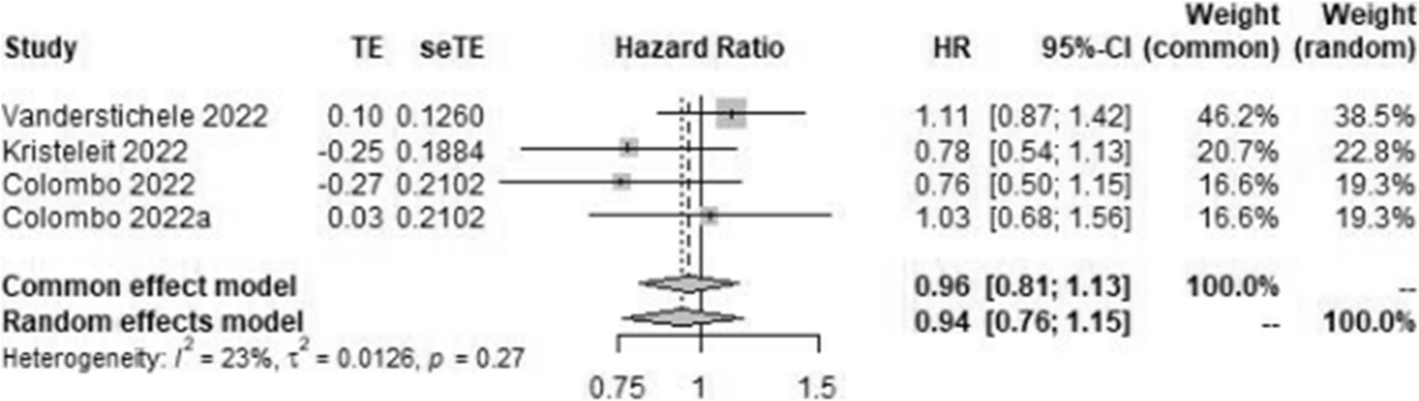
Forest plot showing overall survival in PARP inhibitor arm (case) and chemotherapy (placebo) arm in platinum-resistant ovarian cancer (control)

A total of 14 primary studies were considered for final quantitative analysis (Fig. 1); of these 5 that have been updated, Povedo et al.’s [20] study is the final analysis of SOLO 2, Bannerjee et al.’s [9] study is the updated analysis of SOLO 1, and Swisher et al.’s [22] is the updated analysis of VELIA trial. The study of Lederman 2014 [15] and 2016 [16] are pre-planned retrospective analysis of data based on BRCA status from Lederman 2012 [14]. Wherever available, the updated data has been used. A total of 5363 patients were included; among them, 3513 received PARP inhibitors and 1850 were controlled (receiving placebo or chemotherapy). The baseline characteristics of patients enrolled in different trials are shown in Table 1. Among the 14 studies, 6 studies were on the maintenance therapy after recurrence with PARP inhibitors [10, 12, 14, 17, 21, 25], 3 studies on the first-line maintenance [6, 7, 11] and in 5 studies in relapsed ovarian cancer [13, 18, 19, 22, 23]. Most of the trials were phase 3 except for a few phase 2 trials [12, 14, 18, 24]. All studies used platinum-sensitive ovarian cancer for analysis, while 3 recent studies provided data for the use of PARP inhibitors in platinum-resistant ovarian cancer in subgroup analysis [13, 23, 24]. As there is already established role of PARP inhibitors in platinum-sensitive ovarian cancer, in both first-line and recurrent settings in the HRD population and BRCA-mutant population, subgroup analysis of these groups is not performed separately as it will show similar results. HR of PFS was 0.50 [95% CI 0.40–0.62] favoring the role of PARP inhibitors with 86% heterogeneity seen for all studies included in meta-analysis and those which reported on this outcome (Fig. 2). In the PROC group, HR was 0.94 [95% CI 0.76–1.15], with diamond slightly shifted towards the PARP inhibitors, also favoring PARP inhibitors in platinum-resistant relapsed ovarian cancers; however, it was not statistically significant (Fig. 3).

**Fig. 4:**
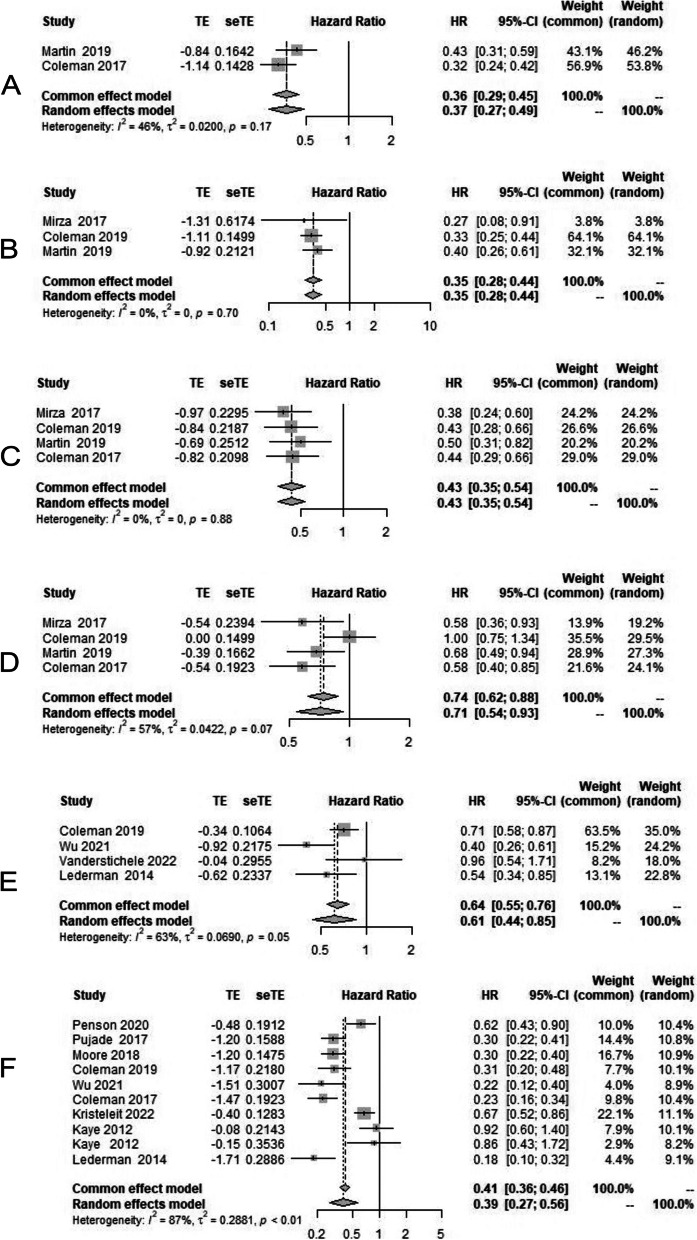
Forest plot showing progression-free survival in various subgroup analysis in PARP inhibitor arm (case) and chemotherapy/placebo arm(control); **A** HRD, BRCA unknown population; **B** BRCAmutated, HRD population; **C** HRD, BRCA wildtype population; **D** Homologous recombination proficient population; **E** BRCA wildtype, HRD unknown population; **F** BRCAmutated, HRD unknown population

Subgroup meta-analysis of data in HRD cohort and BRCA-mutant cohort was carried out by making a total of 6 subgroups in platinum-sensitive ovarian cancer (HRD with BRCA unknown status; HRD with BRCA mutant; HRD with BRCA mutation not detected; HRP group, BRCA mutation absent with HRD status unknown; BRCA mutation). BRCA mutation group includes both BRCA 1 and BRCA 2 mutation detection on germline and/or somatic testing. HRD group includes high LOH group. On analysis, HR of PFS in HRD with BRCA unknown status was found to be 0.37 [95% CI 0.27–0.49] (Fig. 4A), HR of PFS in HRD with BRCA mutant was 0.35 [95% CI 0.28–0.44] (Fig. 4B), and PFS of HRD with BRCA wild type was 0.43 [95% CI 0.35–0.54] (Fig. 4C). In the HRP group, HR of PFS observed was 0.71 [95% CI 0.54–0.93] (Fig. 4D), in patients with BRCA wild type with unknown HRD status, HR of PFS was 0.61 [95% CI 0.44–0.85] (Fig. 4E). All these values were statistically significant and showed benefit of PARP inhibitors. In the last with proven group having role of PARP inhibitors, i.e., BRCA mutation, HR of PFS was 0.39 [95% CI 0.27–0.56] (Fig. 4F).

**Fig. 5:**
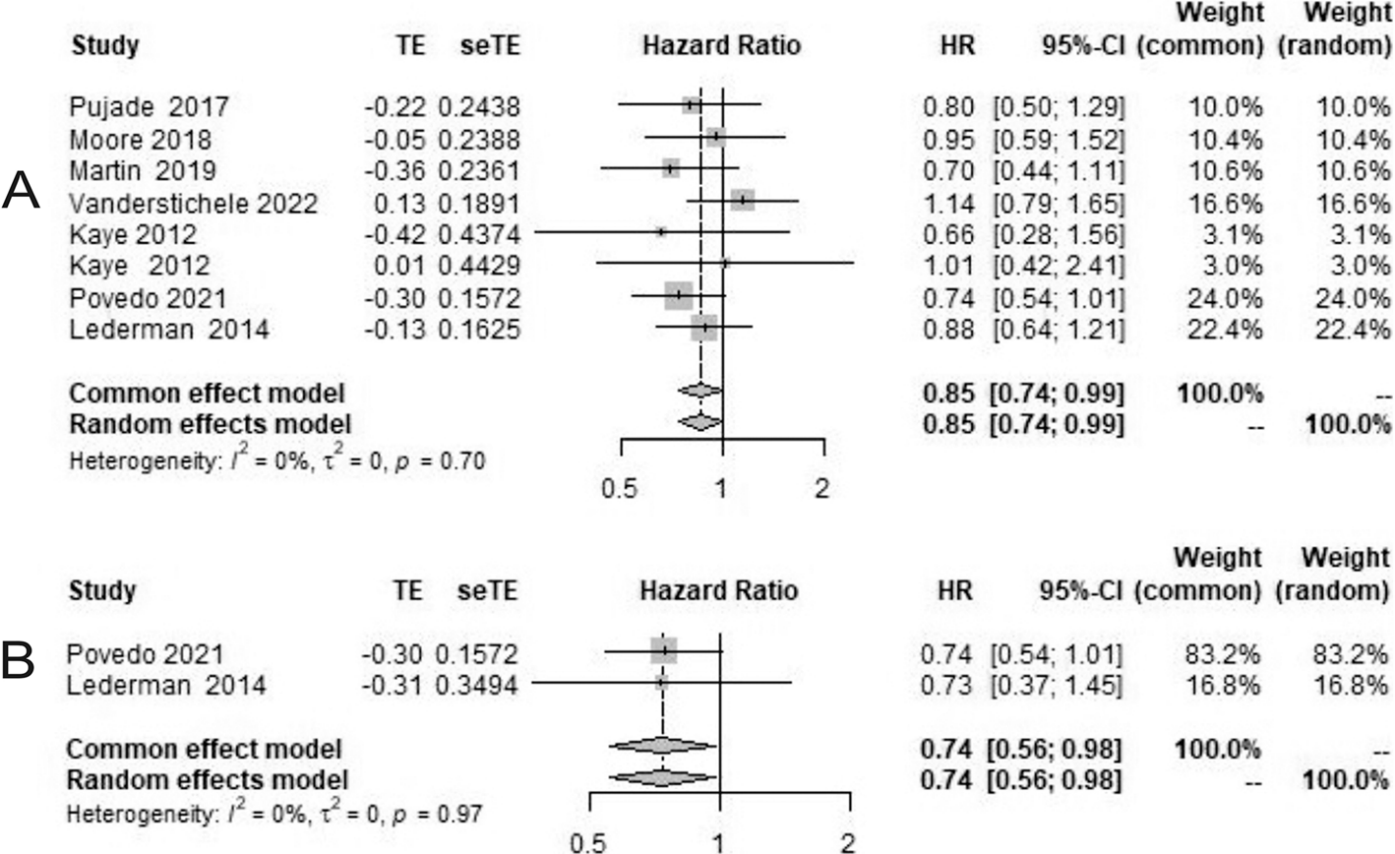
Forest plot showing overall survival in PARP inhibitor arm (case) and chemotherapy/placebo arm in platinum-resistant ovarian cancer (control); **A** overall population; **B** BRCAmutated population

On the analysis of overall survival, in intention to treat population among all groups irrespective of use of PARP inhibitors in first line or recurrent settings (time from randomisation to death) HR was 0.85 [95% CI 0.74–0.99] (Fig. 5A), while in BRCA-mutant population, the HR of OS was 0.74 [95% CI 0.56–0.98] (Fig. 5B).

**Fig. 6:**
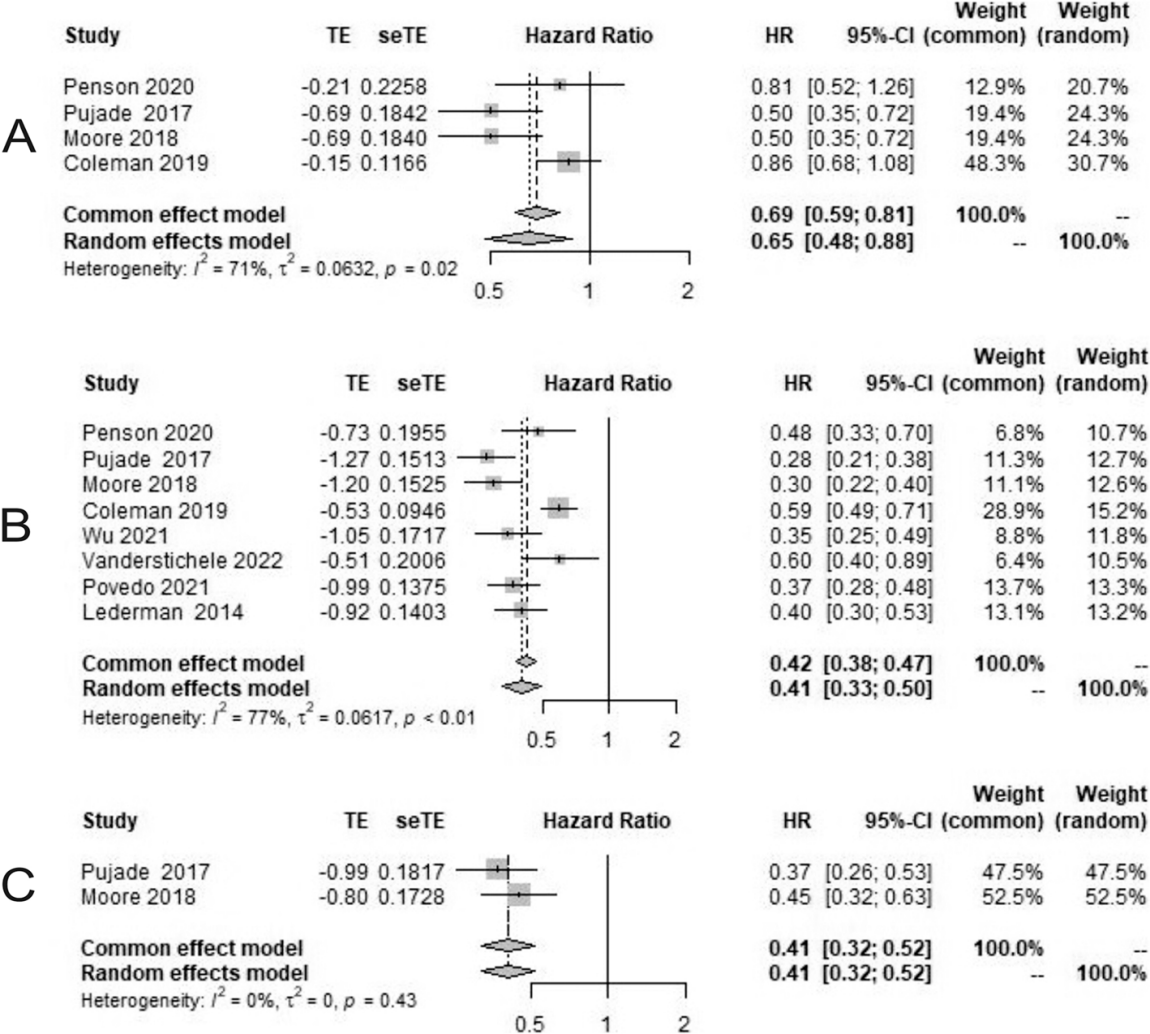
Forest plot showing secondary endpoints in overall population in PARP inhibitor arm (case) and chemotherapy/placebo arm in platinum-resistant ovarian cancer (control); **A** Second progression-free survival (PFS 2); **B** Time to first subsequent therapy (TFST); **C** Time to second subsequent therapy (TSST)

On analysis of our secondary endpoint, second progression-free survival (i.e., time from random assignment to second progression or death), HR was 0.65 [95% CI 0.48–0.88] (Fig. 6A), which is statistically significant. Time from randomization to the first subsequent therapy or death (TFST) HR is 0.43 [95% CI 0.35–0.52] (Fig. 6B), favoring PARP inhibitors. The time from randomization to second subsequent therapy or death (TSST), the HR of PFS was 0.41 [95% CI 0.32–0.52] (Fig. 6C).

## Discussion

In this study, overall results show a statistically significant role of PARP inhibitors in epithelial cancer ovary in comparison to control group (placebo/ chemotherapy). In HRD cohort and in BRCA mutation cohort, there is a significant improvement in PFS as shown by many randomised control trials [8, 38], and the mechanism of action of PARP inhibitors in BRCA mutation cohort is well known [4]. PARP inhibitors and platinum-based chemotherapy are both genotoxic agents and both exploit the DNA repair pathway. Hence, sensitivity to one agent confers sensitivity to other and this is the rationale for using PARP inhibitors in platinum sensitivity [39]. This is seen in all RCTs, and in addition, in the ARIEL 4 trial [13], BAROCCO trial [24], and CLIO trial [23], response is also seen in platinum-resistant relapsed ovarian cancer. CLIO trial also includes wild type BRCA population with BRCA-mutated population. Although our meta-analysis showed HR of PFS in PROC is 0.94, showing slight response; in CLIO trial, the response rate of 17.9% in overall cohort and 38.9% in BRCA-mutated cohort was seen compared to chemotherapy in platinum-resistant ovarian cancer [23].

On subgroup analysis, in non-BRCA-related HRD population, an improvement in PFS like that in PRIMA trial was seen [11]. Clinical benefit of Niraparib in maintenance therapy of primary ovarian cancer was seen in all patients including both HRD and HR proficient populations, PARP inhibitors provided a sustained progression-free survival, beyond chemotherapy, irrespective of BRCA mutation. In patients with high chances of relapse like those with partial response to platinum-based chemotherapy, Niraparib provides significantly prolonged progression-free survival. Likewise, in NOVA trial [17], similar effect of Niraparib maintenance was seen in the platinum-sensitive recurrent ovarian cancer in all the subgroups irrespective of BRCA mutation. In HRD population with wild type BRCA, there was also similar risk of disease progression.

In HRP group, where DNA repair mechanisms are intact, PARP inhibitors have a role in prolonging progression-free survival as seen in various studies. In HR-proficient pathway, the mechanism of synthetic lethality which is described in BRCA mutated and BRCAness population does not happen because of intact DNA repair pathway [4]; hence, apart from DNA repair mechanism, other mechanisms are also working, e.g., promotion of cytotoxic effects by agitating DNA replication by formation of destabilizing DNA replication forks and by increasing replication fork speed, causing DNA replication stress and cell death [40], gene transcription, ribosome biogenesis, and immune activation [11]. Our meta-analysis also suggests there is an improvement in PFS in HRP population or BRCA wild type cohort.

Overall survival data is mature for very few studies; in SOLO 2 [21] trial in platinum-sensitive recurrent ovarian cancer, median OS was 51.7 months, and this was not statistically significant when compared with placebo, but it is clinically meaningful because the difference was 12.9 months (HR 0.74; CI- 0.56–0.98; *P* = 0.05). In platinum-sensitive ovarian cancer in primary settings, González-Martín et al. [11] showed probability of survival at 2 years is 84% in Niraparib group and 77% in the placebo group, with HR = 0.7; CI 0.44–1.11), and in SOLO1 trial [7], the probability of survival after 3 years of Olaparib showed 84 versus 80% in Olaparib versus placebo (HR − 0.95; CI, 0.60–1.530). The pooled results of meta-analysis showed that overall survival is slightly better in PARP inhibitors group than placebo, but the effect of PARP inhibitors is more prolonged in BRCA mutated population and there is no detrimental effect of survival.

In the SOLO series, it was seen that second progression was subsequently prolonged after Olaparib either in the first-line maintenance, recurrent maintenance, or in treatment with Olaparib in platinum refractory cancer, suggesting that Olaparib did not disable another chance for the patient to benefit from subsequent therapy [7, 19, 21], i.e., from oral PARP inhibitor therapy to subsequent intra venous chemotherapy [41]. Our meta-analysis also show a significant improvement in the second progression with the PARP inhibitors.

TFST and TSST are clinically meaningful endpoints in assessment of disease recurrence and restarting of the first and second subsequent therapy, suggesting sustained PFS benefit and thus is a signature for overall survival benefit [41]. In this meta-analysis, TFST is highly significant showing significant survival advantage with the use of PARP inhibitors.

The strength of this meta-analysis is that it is the first meta-analysis of its kind providing pooled survival data analysis for HRP population and BRCA wildtype population. We have also analyzed the survival data for use of PARP inhibitors in platinum-resistant ovarian cancer which was also not mentioned in previous published meta-analysis. Analysis of overall survival data was also unique for our study as it was not provided by any previous clinical trials. We have also analyzed the PFS data as given by updated randomized control studies.

The limitations of our study are that the analysis was done on study level data rather than individual patient data. We have not provided separate data for PARP inhibitors in the first line and recurrent settings, but this type of analysis is already done in the previously published meta-analysis [8]. The role of PARP inhibitors is well established in HRD and BRCA-mutant population [38], but there is a need for further studies on expanding the role of PARP inhibitors in HRP population and platinum-resistant cohort.

The results of this meta-analysis suggest that PARP inhibitors have a role in epithelial cancers of ovary, both in the platinum-sensitive and platinum-resistant ovarian cancers in the first line as well in the recurrence. The results further suggest that in the future, PARP inhibitors can be used in epithelial ovarian cancer without HR and BRCA testing as they provide meaningful clinical benefits in terms of improved PFS in all the subgroups.

## Supplementary Information

**Additional file 1.** Supplement file 1- Cochrane risk of bias tool for the included studies.

**Additional file 2.** Package meta R used for data analysis.

**Additional file 3.** PRISMA checklist for reporting of meta analysis.

## Data Availability

All data generated and analyzed has been provided in the manuscript or in the supplementary file section.
