# Supplementary figures and images for: Role of PARP inhibitors beyond BRCA mutation and platinum sensitivity in epithelial ovarian cancer: a meta-analysis of hazard ratios from randomized clinical trials
Source: World J Surg Oncol. 2023 May 23;21:157. doi: 10.1186/s12957-023-03027-4 (PMC10204292; doi:10.1186/s12957-023-03027-4)

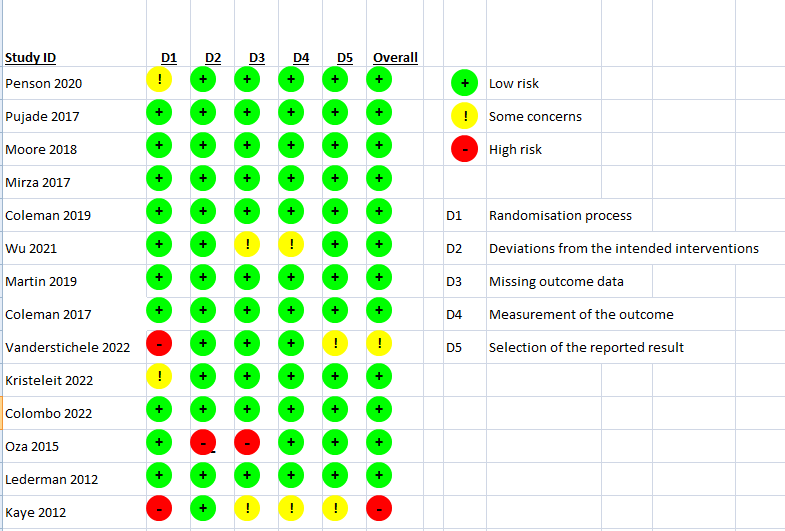

Supplement: Supplementary file 1 — Additional file 1. Supplement file 1- Cochrane risk of bias tool for the included studies. [file 12957_2023_3027_MOESM1_ESM.png]
